# Identification of significantly mutated subnetworks in the breast cancer genome

**DOI:** 10.1038/s41598-020-80204-5

**Published:** 2021-01-12

**Authors:** Rasif Ajwad, Michael Domaratzki, Qian Liu, Nikta Feizi, Pingzhao Hu

**Affiliations:** 1 Department of Biochemistry and Medical Genetics, University of Manitoba, Winnipeg, MB Canada; 2 Department of Computer Science, University of Manitoba, Winnipeg, MB Canada; 3 Research Institute in Oncology and Hematology, CancerCare Manitoba, Winnipeg, MB Canada

**Keywords:** Network topology, Prognostic markers, Genetics research, Breast cancer

## Abstract

Recent studies showed that somatic cancer mutations target genes that are in specific signaling and cellular pathways. However, in each patient only a few of the pathway genes are mutated. Current approaches consider only existing pathways and ignore the topology of the pathways. For this reason, new efforts have been focused on identifying significantly mutated subnetworks and associating them with cancer characteristics. We applied two well-established network analysis approaches to identify significantly mutated subnetworks in the breast cancer genome. We took network topology into account for measuring the mutation similarity of a gene-pair to allow us to infer the significantly mutated subnetworks. Our goals are to evaluate whether the identified subnetworks can be used as biomarkers for predicting breast cancer patient survival and provide the potential mechanisms of the pathways enriched in the subnetworks, with the aim of improving breast cancer treatment. Using the copy number alteration (CNA) datasets from the METABRIC (Molecular Taxonomy of Breast Cancer International Consortium) study, we identified a significantly mutated yet clinically and functionally relevant subnetwork using two graph-based clustering algorithms. The mutational pattern of the subnetwork is significantly associated with breast cancer survival. The genes in the subnetwork are significantly enriched in retinol metabolism KEGG pathway. Our results show that breast cancer treatment with retinoids may be a potential personalized therapy for breast cancer patients since the CNA patterns of the breast cancer patients can imply whether the retinoids pathway is altered. We also showed that applying multiple bioinformatics algorithms at the same time has the potential to identify new network-based biomarkers, which may be useful for stratifying cancer patients for choosing optimal treatments.

## Introduction

The accumulation of somatic genetic mutations, such as single nucleotide variants and copy number alterations (CNAs), drives cancer progression^1^. Somatic CNAs are changes in the copy numbers of a DNA sequence that arise during the process of cancer development. They have been found to be prevalent in breast cancer^2^. Genes in the CNA regions that have changes to the chromosome structure in the form of gain or loss in copies of DNA segments, if mutated, can create abnormal proteins with different functions than a normal protein, which can lead to uncontrollable growth of cancer cells.

Not all mutations cause damage to the human cells: depending on the location of the mutations in the gene, the alteration can make no difference or can even be beneficial. In fact, of all the mutations that occur in human genome, only a few, known as driver mutations, can drive the cancer development^3^.

One of the main challenges in cancer genomics research is to identify the driver mutations. Next generation sequencing and microarray techniques have been popular in characterizing driver mutations. These technologies can analyze cancer genomes for large cohorts of patients in a timely way, which allows more differentiation between driver mutations and passenger mutations than traditional Sanger sequencing technology. Although these are significant advancements, the development and application of new computational methods to analyze and characterize the vast amount of CNA data is still far behind the development of the technologies to generate the data^4^.

One of the major approaches to identify genes with driver mutations is to find genes with a significantly higher mutation frequency or recurrently mutated genes in a collection of cancer patients. For example, The Cancer Genome Atlas (TCGA) study^5^ applied this method to 91 glioblastoma (GBM) patients and identified eight significantly mutated genes with false discovery rate (FDR) smaller than 0.001. In particular, they observed that the TP53 gene is mutated in approximately 38% of the patients. Another TCGA study^6^ examined 316 high-grade serous ovarian cancer patients and identified around 302 TP53 mutations. However, this approach is challenging. Although some cancer genes have higher mutation frequency rates (e.g., TP53) than non-cancerous genes, most of them have much lower mutation frequencies^7^.

However, due to the spatial heterogeneity of cancer genomes, studying individual mutated genes is not sufficient to understand the mechanism of cancer. The single gene test sometimes fails to find other significant genes that are responsible for driver mutations^1^. While the main reason behind the failure can be an insufficient number of patients, individual gene interactions that affect biological function may also affect results. In particular, when the specific functions of the genes are altered due to mutation, the interaction between mutated genes can reveal information related to the mutation that is necessary for understanding the progression of cancer. Therefore, instead of a single gene, driver mutations can target groups of genes, which can be broadly defined as gene clusters or subnetworks in networks or pathways^8,9^.

Compared to analyzing genetic mutation data at single gene level, pathway and network analyses can extract more information as these methods deal with multiple genes in the same pathway or network, so the probability that a molecular event will pass the statistical threshold is increased and the number of hypotheses tested is reduced^10^. Another benefit of pathway analysis is that results obtained for different related datasets can be compared easily, as pathway information can ensure the interpretation of the data is done in a common feature space^11^. This approach to test associations between mutation and phenotype at the pathway level has been implemented in different studies^12–14^. A recent study^15^ conducted a pathway-level analysis to predict the overall survival of urothelial cancer patients. They found that 35 out of 103 samples had mutations in at least one of TP53 and PIK3CA, which consists of 16% and 9% of the total number of mutations identified in the study. The authors also found that around 65% of the patients had CNA mutations.

Pathway level analysis can increase the statistical power to identify significantly mutated pathways in specific cancers and has better biological interpretation. However, the approach of identifying pathways being mutated in large numbers of patients has its limitations too, because only existing pathways are considered, ignoring the topology of the pathways. Moreover, the pathways are analyzed in isolation but they interact in larger networks, which may neglect many groups of interacting genes that are not in known pathways but have significant association with clinical phenotypes^3^.

In recent years, methods to identify mutated subnetworks among cancer genomes have been introduced. Cerami et al.^16^ proposed a network-based approach based on the hypothesis that cellular networks are modular and have inter-connected proteins that perform specific biological functions. The authors used a unified molecular interaction network consisting of both protein–protein interactions and pathways to perform an integrated network analysis for identifying candidate driver mutations. Their approach was a combination of sequence mutations and CNAs.

Vandin et al.^1^ introduced another approach to find subnetworks by considering that mutations in the subnetwork are correlated with the clinical parameter of survival time of patients. They presented an algorithm called HotNet to identify significantly mutated subnetworks determined by the mutation frequency of individual genes along with the interactions between them. They considered the mutation as a source of heat on the network and extracted the ‘hot’ nodes. The significance of the subnetworks was calculated using both the topology of the networks and the frequency of mutation of the genes. Leiserson et al.^17^ introduced HotNet2, a modified version of the HotNet algorithm. They used this updated algorithm to analyze a Pan-Cancer dataset of 3281 samples from 12 cancer types. The authors identified significantly mutated subnetworks with known pathways such as TP53, RTK, and PI3K. The HotNet and its variant of HotNet2 demonstrated the advantages of using network approaches to identify mutated subnetworks with clinical prognosis significance. However, the authors noted that some genes with very high individual mutation scores were absent from the network analysis results and stated that this is due to a lack of data as well as false negatives in the analyzed data.

One key limitation of the subnetwork-based approaches is that they do not assign the same mutated genes into different subnetworks although overlapping subnetworks are possible. Nepusz et al. developed a network-based clustering algorithm, called ClusterOne^18^, which can identify overlapping clusters in protein interaction networks. This motivates us to apply the two well-established network-based clustering algorithms, HotNet2 and ClusterOne, to analyze breast cancer CNA mutation data. We measure the mutation similarity of gene pairs and use the two algorithms to identify significantly mutated subnetworks and test the association of the genes in the subnetworks with breast cancer patients’ survival.

## Materials and methods

### Data collection and filtering

Identification of significantly mutated subnetworks in the breast cancer genome requires both patient-specific mutations and gene interaction networks. For patient-specific mutation data, we used CNA data obtained from the METABRIC (Molecular Taxonomy of Breast Cancer International Consortium)^19^. The CNA calls were identified from approximately 2000 clinically annotated primary fresh frozen breast cancer specimens along with a portion of normal specimens from different North American and European tumor banks. Initially, a set of 997 samples including paired DNA and RNA profiles were analyzed. The authors referred to these 997 female patient data as the ‘Discovery set’. A second group of 995 samples was referred to as the ‘Validation set’, for which the paired DNA and RNA profiles were not available during the initial study and collected at other time periods. The purpose of the Validation set was to test the reproducibility of the clinical outcome associations.

In the data sets, there were five discrete somatic states: HOMD, HETD, NEUT, GAIN and AMP. We mapped these five states to three CNA states: HOMD and HETD were broadly called ‘loss’ and GAIN and AMP were broadly called ‘gain’ and NEUT was represented by ‘neutral’. The CNA calls were represented for gain, loss and neutral as 1, − 1 and 0 respectively. We filtered out the data where the CNA length was 5 kilobase (KB) or longer and the number of probes was ten or more for the remaining analyses. In the Discovery set, 131,956 calls (38,647 CNA loss and 93,309 CNA gain) remained after the filtering. In the Validation set, 137,896 calls (42,824 CNA loss and 95,072 CNA gain) remained.

The gene interaction network data was taken from^20^, which included 141,296 interactions between 13,460 human proteins. Interactions from gene expression data were not included. The interactions are primarily protein–protein interactions (PPI), but the dataset also included other types of physical interactions such as regulatory interactions, protein complexes from the comprehensive resource of mammalian protein complexes (CORUM) database and signaling interactions^21^. The authors treated the interactions as an undirected network.

### Methods

**Figure 1:**
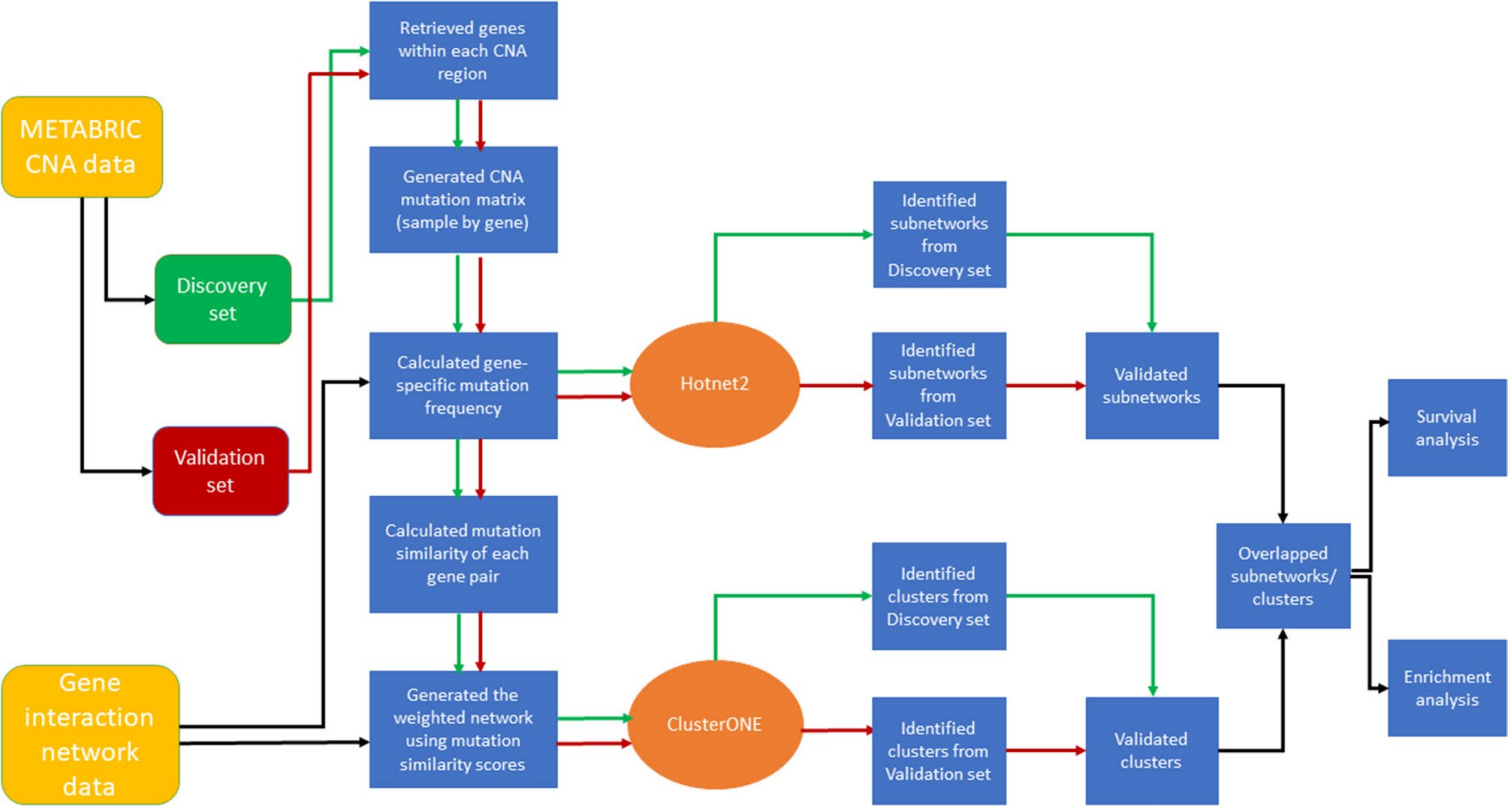
Flowchart to identify significantly mutated gene subnetworks. HotNet2 and ClusterONE were used to identify significantly mutated gene subnetworks or clusters from a curated gene interaction network and CNA mutation data in the METABRIC Discovery and Validation sets, respectively. The clinical and biological pathway significance of the overlapping genes in the subnetworks (clusters) identified by both algorithms in both the Discovery and Validation sets were evaluated.

We applied two well-established network analysis approaches to identify the clinically relevant and statistically significantly mutated subnetworks as shown in Fig. 1. Briefly speaking, we first retrieved gene information in each sample-specific individual CNA region; then we calculated the gene-specific mutation frequency and the gene-pair specific mutation similarity score, which, together with the gene interaction network, were passed to the HotNet2 and ClusterONE tools to identify statistically significantly mutated subnetworks and gene clusters, respectively; finally, we evaluated the clinical and pathway significance of the overlapping genes identified in both the mutated subnetworks and gene clusters. These steps are detailed as follows.

### Retrieve CNA-specific genes

**Figure 2:**
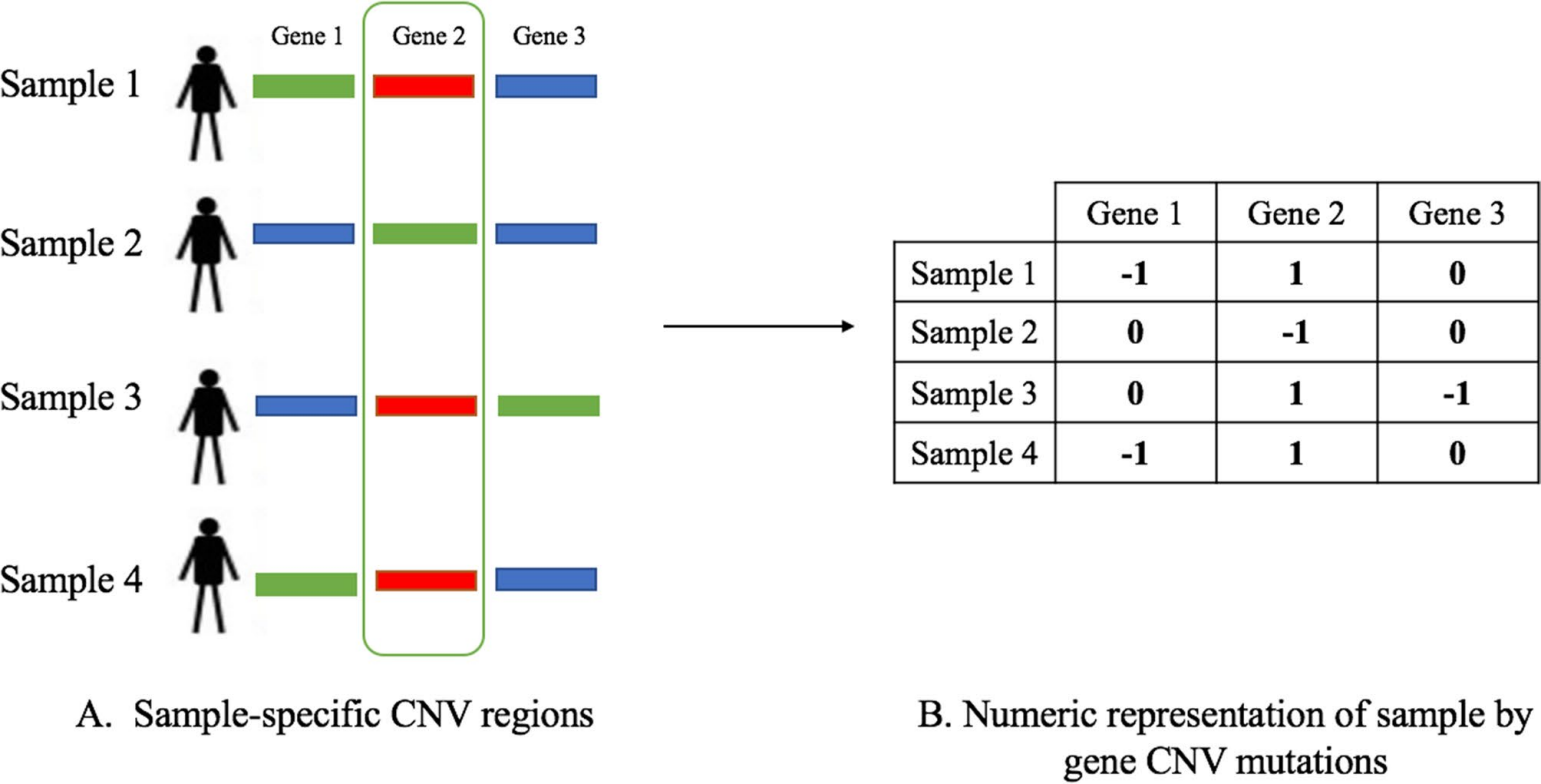
From sample specific CNA regions to gene- and sample-specific CNA mutations. Gene information was retrieved for each sample-specific CNA region (**A**), where regions labeled as blue represent no CNA change, regions labeled as red represent as CNA gain and regions labeled as green represent as CNA loss. The non-mutated genes are expressed as ‘0’ and mutated genes are expressed as ‘1’ for gain and ‘− 1’ for loss, respectively (**B**).

We retrieved gene information for each of the patient-specific CNA regions using the BiomaRt R package^22^ (Fig. 2A). We used the ‘hsapiens_gene_ensembl’ dataset from the ENSEMBL database, which contains human genes. We used the hg19 version of the database to retrieve the CNA-specific genes. The parameters we used are from the filtered CNA data: the chromosome ID, and the start and end locations of the CNAs in the chromosome. After we retrieved the genes for each CNA region, we generated a sample-by-gene CNA mutation matrix, where the rows were the filtered samples and the columns were the genes found in the CNA regions. The gene- and sample- specific CNA mutation matrix was generated by expressing the non-mutated genes as ‘0’ and mutated genes as ‘1’ for gain and ‘− 1’ for loss, respectively (Fig. 2B).

### Calculate gene-specific mutation frequency

After generating the mutation matrix, we calculated the gene-specific mutation frequency. The main motivation behind calculating gain and loss frequencies was to have a measure for gene-specific mutation score. We used this mutation frequency as the ‘heat score’, which is required to run the Hotnet2 software, as described below.

The mutation frequency for each gene *i* was calculated as:

1 $$f_{ig} = \frac{{n_{g} }}{{N_{s} }}$$

2 $$f_{il} = \frac{{n_{l} }}{{N_{s} }}$$

Here $N_{s}$ denotes the total number of samples in the dataset, whereas $n_{g}$ and $n_{l}$ are the total number of CNA gains and CNA losses of gene *i* in all the samples, respectively. $f_{ig}$ and $f_{il}$ are the mutation frequencies of CNA gain and loss in gene *i*, respectively.

### Calculate gene-pair specific mutation similarity

Since our aim is to identify subnetworks and clusters with genetic defects in the gene interaction network we collected, which is unweighted, we need to first measure the genetic defects at gene-pair level. To do this, we calculated the pairwise gene mutation similarity in the gene interaction network by taking the genetic mutation frequencies into account. We used this measure of gene-pair specific mutation score as the weight for each interaction in the network. This similarity score for each pair is used as an input to ClusterONE, as described below. The original ClusterONE algorithm had a constant weight (= 1) for all the interactions, and the authors stated that using a weighted approach may yield improved results^18^.

For simplicity, we used the cosine similarity^23^ and Eqs. (1) and (2) to measure the gene-pair mutation similarity:

3 $$sim\left( {i,j} \right) = \frac{{\mathop \sum \nolimits_{{k \in \left\{ {g,l} \right\}}} f_{ik} f_{jk} }}{{\mathop \sum \nolimits_{{k \in \left\{ {g,l} \right\}}} \left( {f_{ik} } \right) ^{2} \mathop \sum \nolimits_{{k \in \left\{ {g,l} \right\}}} \left( {f_{jk} } \right) ^{2} }}$$

The similarity measure $sim\left( {i, j} \right)$ for genes $i$ and $j$ is defined by mutation frequency $f$. In our case, we have two types of mutation frequencies: gain ($k = g$) and loss ($k = l$). For our work, we treated the gene-pair similarity as a network edge weight. As the gene interaction network we obtained from^20^ is unweighted, we have assigned the gene similarity to the edges of the network.

### Identify significantly mutated subnetworks

We used two different algorithms to identify significantly mutated subnetworks from our collected breast cancer CNA data and the gene interaction network. HotNet2 identifies mutated subnetworks of a genome-scale interaction network^17^, while ClusterONE identifies clusters or groups of interacted genes in a gene interaction network^18^. We briefly discuss these two algorithms.

#### HotNet2

HotNet2 (HotNet diffusion oriented subnetworks) identifies subnetworks that are mutated more frequently than the general rate of mutation by chance. The authors used an insulated heat diffusion approach, which not only considers the mutation score for each gene, but also leverages the topology of interactions between the genes. The inputs to HotNet2 are: a heat score vector $h$ that contains the mutation score for each gene, and an unweighted graph $G = \left( {V, E} \right),$ where each node $v \in {\text{V}}$ corresponds to a gene/protein and each edge $e \in {\text{E }}$ corresponds to an interaction between the corresponding genes/proteins. In the first step, the algorithm performs ‘heat diffusion’ to extract the local topology of the interaction network. At each iteration, the nodes (genes) in the network send and collect heat from the neighboring nodes. The authors define an insulating parameter $\beta$, which denotes the fraction of the heat retained by each node at each iteration. The iterations terminate when all nodes in the graph reach equilibrium. HotNet2 identifies strongly connected components in the network and returns a list of subnetworks, each containing at least *k* genes for a parameter *k*. The statistical significance of the returned list of subnetworks is then calculated for the number of subnetworks with at least *k* genes that are returned and the false discovery rate for the subnetworks are also estimated. In our study, the gene-specific mutation score $h$ is calculated to be equal to the mutation frequency $f_{i} \left( {f_{i} = f_{ig} + f_{il} } \right)$.

#### ClusterONE

The ClusterONE (Clustering with overlapping neighborhood expansion) algorithm uses a greedy growth process to find groups of genes or proteins (here we treat gene and protein as interchangeable terms) with high cohesiveness in a gene interaction network. The authors generalized two structural properties of a gene module or protein complex represented by a subgraph to define cohesiveness for each group: the interaction between its subunits and the separation from the rest of the network. For a group of proteins $P$, the cohesiveness $C\left( P \right)$ is defined by:

4 $$C\left( P \right) = \frac{Total\; weight\;for\;internal\;edges}{{Total\;weight\;for\;internal\;edges + total\;weight\;for\;boundary\;edges + p}}$$

The term ‘internal edges’ is used to define the edges that have both endpoints with the given group and ‘boundary edges’ are the edges that have a connection with the rest of the network. The constant $p$ is a penalty term to model the uncertainty in the data.

The ClusterONE algorithm has three main steps. First, the algorithm follows a greedy approach to select the protein which has the highest degree as the first seed, and starts to grow a cohesive group from the initial seed based on (4). While selecting the next seed, the algorithm considers all the proteins that are not currently included in any other networks (e.g. pathways or protein complexes). Then the node with the highest degree is taken again. This step continues until there are no more proteins left to consider. The growth process ensures that any node from the group can be removed in later iterations if necessary. This includes the initial seed node. The seed node is selected as an outlier if it is not included in the final group. This means that the node will not be included in any of the clusters.

In the next step, highly overlapping pairs are merged based on the optimal cohesive groups. The authors merge pairs of groups which has an overlap score ($\omega$) larger than 0.8. For two protein sets $X$ and $Y$, the authors defined $\omega$ as:

5 $$\upomega \left( {X,Y} \right) = \frac{{\left| {X \cap Y} \right|^{2} }}{{\left| { X } \right|\left| { Y} \right|}}$$

The authors state that the mergings may be performed one after another or concurrently. For the first approach, the problem is that the overlap scores need to be recalculated in each iteration, after each merging occurs. To avoid this problem, ClusterONE uses the concurrent approach. From a collection of cohesive groups, ClusterONE constructs an overlap graph. Each node in the overlap graph represents a cohesive group in the original graph, and two nodes are connected in the overlap graph if the overlap score is more than the selected threshold ($\upomega \ge 0.8)$. Nodes that have a direct (one-to-one) or indirect (via paths of edges) connection are then merged to convert them into subnetworks (e.g. pathways or protein complexes). If a node does not have an edge, no merging is done and it is promoted to a subnetwork candidate.

In the final step, the algorithm discards complexes which have a density below a particular threshold δ. In our study, we consider the gene network to be a weighted graph and the weight of each gene pair is the gene-pair mutation similarity $sim\left( {i, j} \right).$ The cluster-specific p-value is calculated based on an one-sided Mann–Whitney U test performed on the in-weights and out-weights of the vertices in the cluster. To adjust the p-values from the network analysis, we adjusted the p-values using Benjamini–Hochberg procedure implemented in R function p.adjust.

### Parameters used to run HotNet2 and ClusterONE

HotNet2 runs in four consecutive steps: The first step is to create an influence matrix that defines an "influence score" for each gene pair in the network based on known gene interactions and a heat diffusion process. The second step is heat score generation. In our case, the heat scores were directly calculated from the mutation score. The third step is delta selection, which uses permutated data sets to select thresholds that should be used for edge weight removal in the final step. The output of this step includes a list of selected thresholds for each permutation test. We took the median of the deltas across all permutation tests we performed. The final step identifies the mutated subnetworks based on the influence matrix and heat score, removing edges with weight less than delta, and extracting the resulting connected components. This step does not need any additional parameters. The parameters we used in each of the steps are shown in Additional File 1: Table S1.

To run ClusterONE, we only need a weighted network. We did not change any additional parameters provided by the software.

### Validating results using discovery and validation sets

We first obtained the subnetworks identified from the Discovery and Validation datasets by running the HotNet2 software step by step using the parameters described in Additional File 1: Table S1, respectively. The validation of the mutated subnetworks is done by finding the mutated subnetworks from the Validation set, which have the overlap score threshold at least 50% with the mutated subnetworks identified from the Discovery set. The clusters identified by running ClusterONE from both the Discovery and Validation sets were also validated in the same way.

### Overall survival analysis

To identify potential network biomarkers for breast cancer prognosis, we evaluated the association of mutation patterns of the genes in the identified significantly mutated subnetworks from HotNet2 and clusters from ClusterONE with breast cancer survival. To do this, for a given significantly mutated subnetwork or cluster, we first extracted a submatrix from the mutation influence matrix generated previously that included the genes that we found in the identified subnetwork or cluster. Then, for each gene *i* in the subnetwork or cluster, we have a gene-specific mutation frequency $f_{i} \left( {f_{i} = f_{ig} + f_{il} } \right)$ as shown in (1) and (2), so we can calculate the sample-specific mutation score as:

6 $$p_{j} = \mathop \sum \limits_{i = 1}^{G^{\prime}} f_{i} \cdot |v_{ji} |$$

Here $p_{j}$ is the mutation score for sample *j*, $G^{\prime}$ is the number of genes in the subnetwork or cluster, $f_{i}$ is the mutation frequency score for the gene $i$, and $v_{ji}$ is the copy number alteration status of gene *i* in sample *j* (see Fig. 2B). For example, from the generated mutation matrix shown in Additional File 2: Table S2, we can calculate the sample-specific weighted mutation score for sample 1 as $p_{1}$ = (0.3 × 1) + (0.38 $\times \left| { - 1} \right|$) + (0.5 × 1) = 1.18.

We used the product-limit method, also known as the Kaplan–Meier (KM) method^24^, to estimate a survival function. The KM method is a nonparametric technique that uses the exact survival time for each individual in a sample instead of grouping the times into intervals. To perform KM analysis, we categorized the breast cancer patients into a high risk and a low risk groups based on the sample-specific mutation score using R xtile function. We decided the optimal cut-point of the mutation score that results in a minimum *p* value of log-rank tests. The minimum *p* value approach was originally developed by Miller and Siegmund^25^ to dichotomize continuous predictors with binary outcomes, which was later extended to survival outcomes by Mazumdar and Glassman^26^.

To evaluate the robustness of using the sample-specific mutation score to stratify the breast cancer patients into high and low risk groups, we calculated the sample-specific score in each of the identified significantly mutated clusters and subnetworks using a permutation-based approach as follows:

1. For each of the identified clusters and subnetworks, we randomly generated 1000 clusters and subnetworks by permutating the rows of the copy number alteration matrix (rows are genes and columns are samples), which have the same size as the specified cluster or network;
2. For each of the permutated cluster and subnetworks, we calculated sample-specific mutation score using Eq. 6.
3. The KM survival analysis was performed based on the permutated sample-specific score for each of the identified clusters and subnetworks. Finally, the cluster and subnetwork-specific permutation *p* value is calculated as $p_{i}^{perm} = \frac{{1 + \# \{ p_{i}^{permutated\;data} < p_{i}^{real\;data} \} }}{1001},$ where *i* is the given cluster or subnetwork, $p_{i}^{permutated\;data} \;{\text{and }}\;p_{i}^{real\;data}$ are the survival *p* value of cluster or subnetwork *i* based on the its permutated and real data, respectively.

### Pathway enrichment analysis

Using the gene list from each of the identified significantly mutated subnetworks and clusters, we performed the enrichment analysis of gene ontologies (biological processes and molecular functions) and biological pathways (REACTOME and KEGG) using the Enrichr software^27^. The Enrichr contains a diverse and up-to-date collection of over 100 gene set libraries available for analysis and download. It is used to perform pathway enrichment analysis on the identified overlapping genes from both ClusterONE clusters and Hotnet2 subnetworks to identify which pathways are overrepresented in the overlapping genes.

### Expression analysis of the identified genes

For the identified genes from our network analysis, we first evaluated their expression patterns in normal tissues from Genotype-Tissue Expression (GTEx) portal^28^ as well as breast cancer tissue in METABRIC and TCGA data sets. We then examined the association of their gene expression levels with breast cancer patient’s overall survival using both METABRIC and TCGA gene expression data sets. We fitted Cox's proportional hazards (COX-PH) model of the identified genes for TCGA, METABRIC validation, and METABRIC discovery data, respectively. Using the coefficients (*coeff*) extracted from COX-PH models, we then generated a signature as risk score by combining the genes’ expressions (*E*) for the three datasets, respectively, which is calculated based on: Risk score (RS): $RS_{j} = \sum\nolimits_{i} coeff_{i} *E_{i}$, where *i* is the ith gene and *j* is the jth sample. We binarized the expression levels for the combined risk score into a high risk and low risk groups using R xtile function. The survival differences between these two groups for the risk score were assessed by Kaplan–Meier (KM) curves.

### Ethical approval and informed consent

This is not applicable to this study since all the data used in the study are publicly available.

## Results

We first described the clinical characteristics of the Discovery and the Validation data sets and then compared the mutated subnetworks identified from the two data sets using the two algorithms we described in the “Method” section.

### Clinical characteristics

The Discovery and Validation data sets have very similar distributions in age, and expression levels of progesterone receptor (PR) and Her2 (*ERB-B2*) (*p* > 0.05) (Table 1). On the other hand, the two sets have statistically significant differences in PAM50 subtypes, grade, and expression of estrogen (ER) (*p* < 0.05).

**Table 1:** Clinical characteristics.

Characteristic	METABRIC discovery	METABRIC validation	*p*^†^
**Age**	61 (51, 70)*	63 (52,71)	0.2107
**Subtype**			0.0005
Normal	58 (6%)^‡^	144 (15%)	
LumA	454 (46%)	255 (26%)	
LumB	266 (27%)	222 (23%)	
Her2	84 (9%)	153 (15%)	
Basal	118 (12%)	211 (21%)	
**Grade**			0.0095
1	68 (7%)	98 (11%)	
2	407 (42%)	356 (40%)	
3	505 (51%)	444 (49%)	
ER-expr	784 (80%)	712 (72%)	< 0.0001
PR-expr	517 (53%)	517 (52%)	0.9282
Her2-expr	112 (11%)	132 (13%)	0.1940

*For continuous variables (Age), quartiles are presented.

^†^*p* values were determined by Wilcoxon rank sum test for continuous variables and Chi-square test for categorical variables.

^‡^The number of patients in each category and its proportion are presented. In ER-expr, PR-expr and Her2-expr, only the number of positive case are presented.

### Mutation frequency and mutation similarity

Based on the individual-specific CNA positions, we retrieved 18,341 genes in both the Discovery and Validation sets. Based on the gene- and sample-specific mutation matrix (Fig. 2B), the genes with the highest number of CNA mutations were *SLC41A1* and *LEMD1*, both with a CNA gain mutation frequency of approximately 46%. In both of the Discovery and Validation sets, approximately 70% of the genes have a mutation frequency lower than 10%. There are 6.1% and 7.3% of the genes with a mutation frequency higher than 30% in the Discovery and Validation sets, respectively. The gene-specific mutation frequencies were treated as mutation scores in HotNet2 to identify significantly mutated subnetworks.

We calculated the mutation similarity for the gene pairs of the 141,296 gene interactions from the gene interaction network. We divided the interactions into three different groups: high mutation group (interactions with a mutation similarity score at least 0.9), medium mutation group (interactions with a mutation similarity score less than 0.9 but at least 0.5) and low mutation group (mutation similarly scores below 0.5). Based on these thresholds, we found a total of 51,953, 37,251 and 52,092 interactions in the high, medium and low mutation groups, respectively. The gene-pair specific mutation similarities were treated as weights for the gene interaction network in ClusterONE, to identify significantly mutated gene clusters.

### Significantly mutated subnetworks identified by HotNet2

We found a total of 99 and 79 subnetworks that have at least three interacting genes from the Discovery and Validation data sets, respectively. Ten of these subnetworks were identified as significantly mutated subnetworks based on (1) a false discovery rate < 0.1; (2) an overlapping rate (number of overlapping genes divided by the minimum number of genes in either the Discovery or Validation set) being greater than or equal to 50% (Table 2). All of these 10 subnetworks have 3–7 interacting genes.

**Table 2:** Significantly mutated subnetworks identified by HotNet2.

Discovery set		Validation set		No. of overlapping genes	Overlapping subnetwork ID
Subnetwork ID	No. of genes	Subnetwork ID*	No. of genes		
18	5	9	6	5	S1
32	4	6	7	4	S2
38	4	20	5	4	S3
49	4	8	7	4	S4
50	4	21	5	4	S5
79	3	23	4	3	S6
81	3	33	4	3	S7
89	3	16	6	3	S8
93	3	35	4	3	S9
96	3	13	6	3	S10

The subnetworks shown in this table were selected based on (1) adjusted *p* value < 0.1; and (2) the overlapping rate (number of overlapping genes divided by the minimum number of genes in either Discovery or Validation set) being larger than or equal to 50%.

*Subnetworks identified in Validation set with the largest number of overlapping genes for a given subnetwork identified in Discovery set.

### Significantly mutated gene clusters identified by ClusterONE

**Figure 3:**
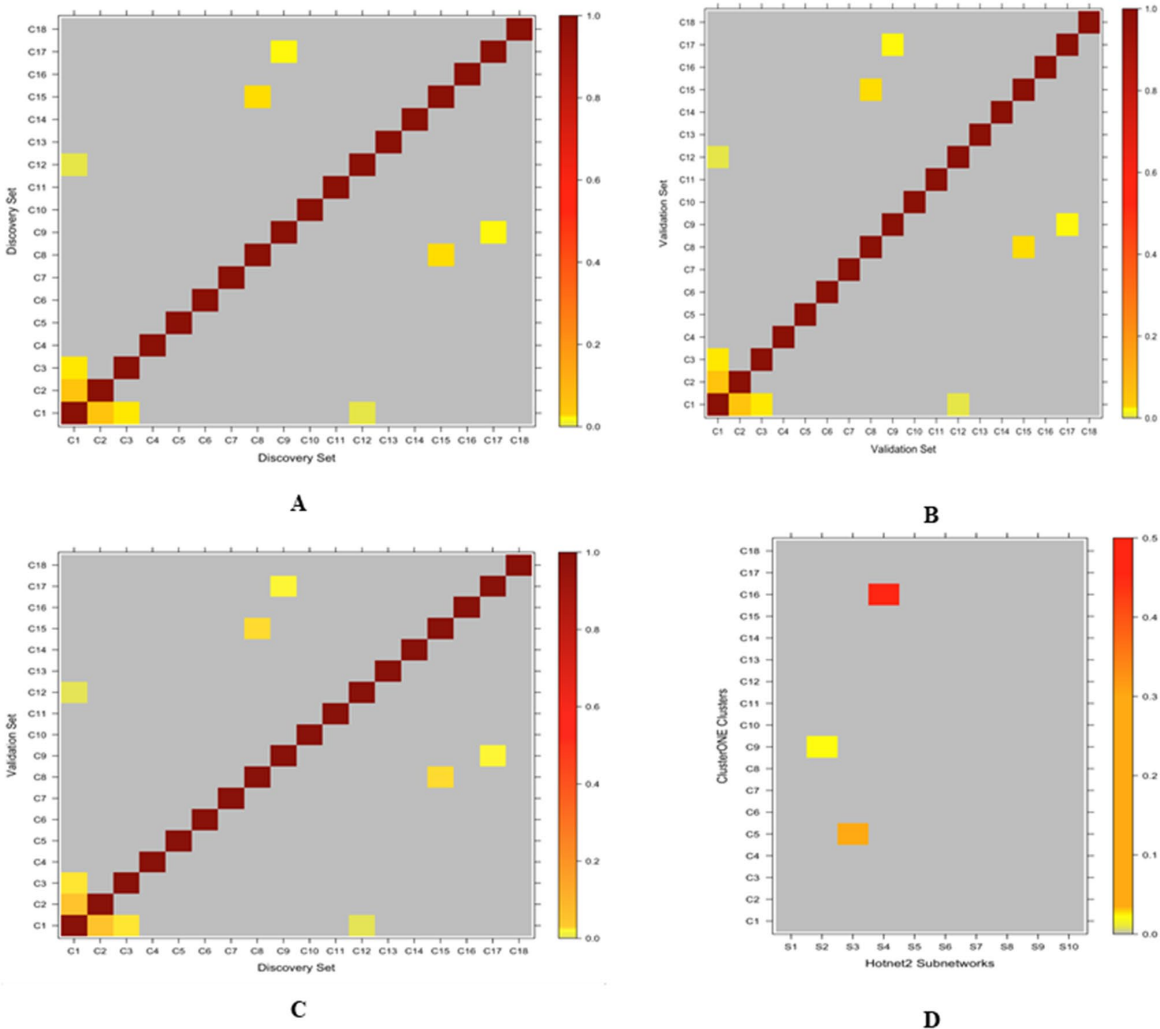
Heatmap of overlapping score for each pair of gene clusters. The overlapping score is defined as the number of overlapping genes divided by the minimum number of genes in either of the pair of gene clusters. The score is represented with the bar in the right, red being the highest overlapping score (1.0) and grey being the least (0.0). (**A**) Gene clusters identified by ClusterOne in the Discovery set; (**B**) Gene clusters identified by ClusterOne in the Validation set; (**C**) Gene clusters identified by ClusterOne in the Discovery set and Validation set; (**D**) The pairs of gene clusters identified by HotNet 2 (X-axis) and ClusterOne (Y-axis). Note: The gene clusters from ClusterOne are the overlapping gene clusters (see Table 3).

We identified 18 significantly mutated gene clusters in both the Discovery and Validation sets from 2242 clusters with cluster size larger than 2 in the Discovery set and 2231 clusters with cluster size larger than 2 in the Validation set, which have (1) an adjusted *p* value < 0.1; and (2) an overlapping rate greater than or equal to 50% (Table 3). Five of the 18 clusters have at least 50 genes. Ten of the 18 clusters have fewer than 30 genes. The heatmaps in Fig. 3A–C, show the overlapping rate between the 18 clusters from the Discovery, Validation and Discovery vs. Validation sets, respectively. It appears that some of the clusters have shared genes (that is, the same gene can be assigned to multiple clusters). For example, cluster C1 has shared genes with clusters C2, C3 and C12 in both the Discovery and Validation sets, respectively. Table 3 shows the number of overlapping genes between the clusters from the Discovery and Validation data sets. It is clear that all of the 18 clusters identified in the Discovery set were validated by the Validation set.

**Table 3:** Clusters identified by ClusterONE in Discovery and Validation sets.

Discovery set				Validation set				No. of overlapped genes
Clus ID	No. of genes in clusters	*p* value (before adjustment)	Adjusted *p* value	Cluster ID	No. of genes in clusters	*p* value (Before Adjustment)	Adjusted *p* value	
C1	275	0	0	C1	280	0	0	269
C2	154	0	0	C2	154	0	0	154
C3	77	0	0	C3	77	0	0	77
C4	58	0	0	C4	59	0	0	58
C5	55	0	0	C5	55	0	0	55
C6	40	0	0	C6	40	0	0	40
C7	24	1.55E−09	4.95E−07	C7	24	1.55E−09	4.94E−07	24
C8	35	9.17E−08	2.57E−05	C8	37	8.36E−08	2.33E−05	33
C9	46	3.34E−07	8.31E−05	C9	45	1.43E−07	3.54E−05	41
C10	19	1.05E−06	2.35E−04	C10	19	9.06E−07	2.02E−04	19
C11	19	1.46E−06	2.96E−04	C11	26	1.01E−06	2.05E−04	19
C12	26	1.58E−06	2.96E−04	C12	51	1.17E−05	2.18E−03	26
C13	13	8.60E−06	6.29E−03	C13	12	5.84E−05	9.3E−03	12
C14	12	3.93E−05	8.73E−03	C14	20	8.97E−05	1.33E−02	12
C15	16	5.84E−05	3.93E−02	C15	16	2.12E−04	2.95E−02	16
C16	8	2.81E−04	5.18E−02	C16	8	3.93E−04	5.15E−02	8
C17	16	3.93E−04	6.04E−02	C17	16	4.24E−04	5.25E−02	16
C18	17	6.03E−04	7.11E−02	C18	17	6.79E−04	7.8E−02	17

The clusters (Clus) shown in this table were selected based on (1) adjusted *p* value < 0.1; and (2) the overlapping rate (number of overlapped genes divided by the minimum number of genes in either Discovery or Validation set) being greater than or equal to 50%.

### Comparison of subnetworks identified by HotNet2 and clusters identified by ClusterONE

**Figure 4:**
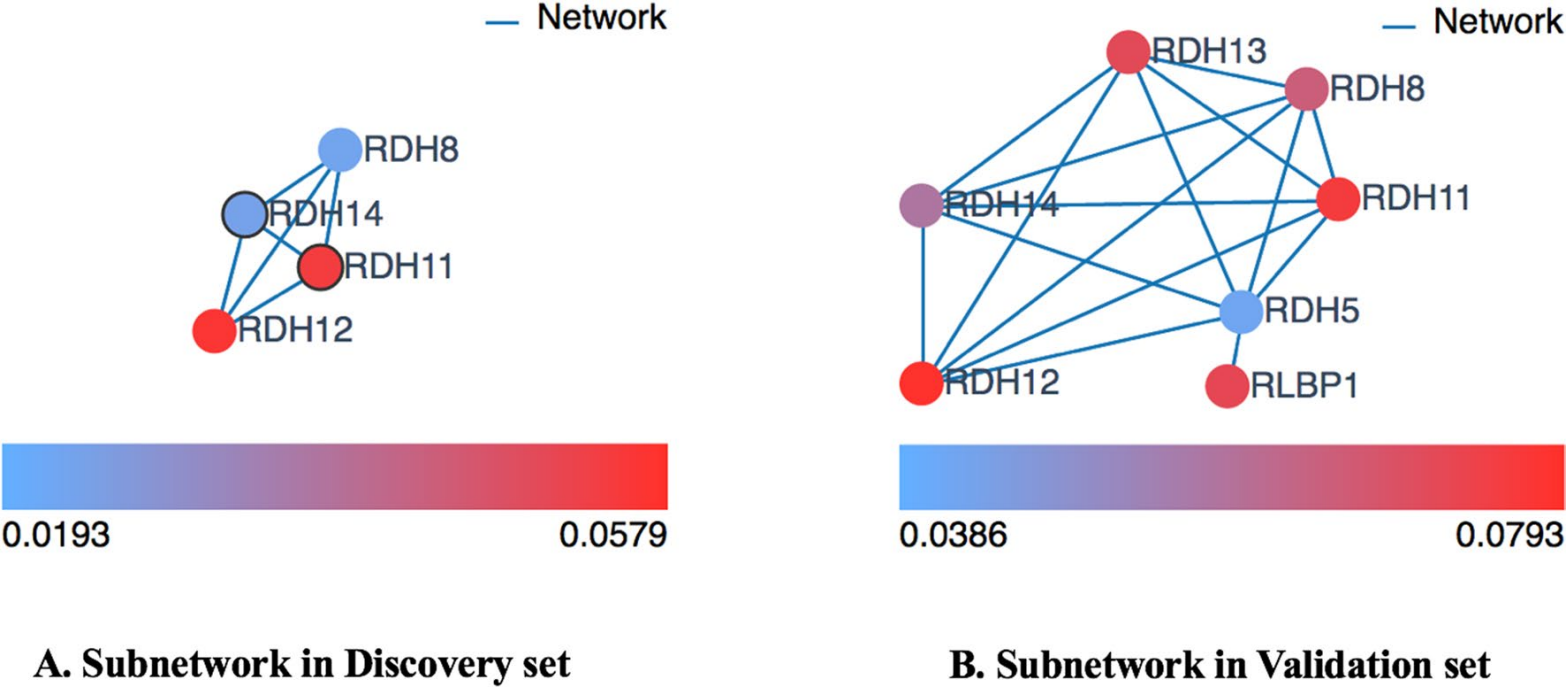
Subnetworks identified by HotNet2. The subnetworks (S4 in Table 2) identified by HotNet2 in the Discovery and Validation sets, respectively. For the identified subnetworks, the heat (mutation level) is shown in the bottom, blue being the least mutated genes and red being the highest mutated gene in the network. (**A**) S4 cluster identified in the Discovery set; (**B**) S4 cluster identified in the Validation set.

We compared the significantly mutated subnetworks (Table 2) identified by Hotnet2 with the significantly mutated clusters (Table 3) obtained by ClusterONE (Fig. 3D). Three significantly mutated subnetworks S2, S3 and S4 have overlapping genes with significantly mutated clusters C5, C9, and C16, respectively, but there is only one significantly mutated subnetwork (subnetwork S4 in Table 2 and cluster C16 in Table 3), which has an overlapping rate larger than 50%. All four genes in the subnetwork S4 are in the cluster C16 with eight genes. The gene cluster C16 identified by ClusterONE includes eight genes *RDH5, RDH8, RDH10, RDH11, RDH12, RDH13, RDH14, SDR16C5* in both the Discovery and Validation sets. The subnetwork S4 identified by HotNet2 includes four genes *RDH8, RDH11, RDH12* and *RDH14* in the Discovery set and seven genes *RDH5, RDH8, RDH11, RDH12, RDH13, RDH14* and RLBP*1* in the Validation set. As shown in Fig. 4, the two genes *RDH11* and *RDH12* are highly mutated in Discovery set (Fig. 4A) and the six genes *RDH8, RDH11, RDH12, RDH13, RDH14* and RLBP*1* are highly mutated in Validation set (Fig. 4B).

**Figure 5:**
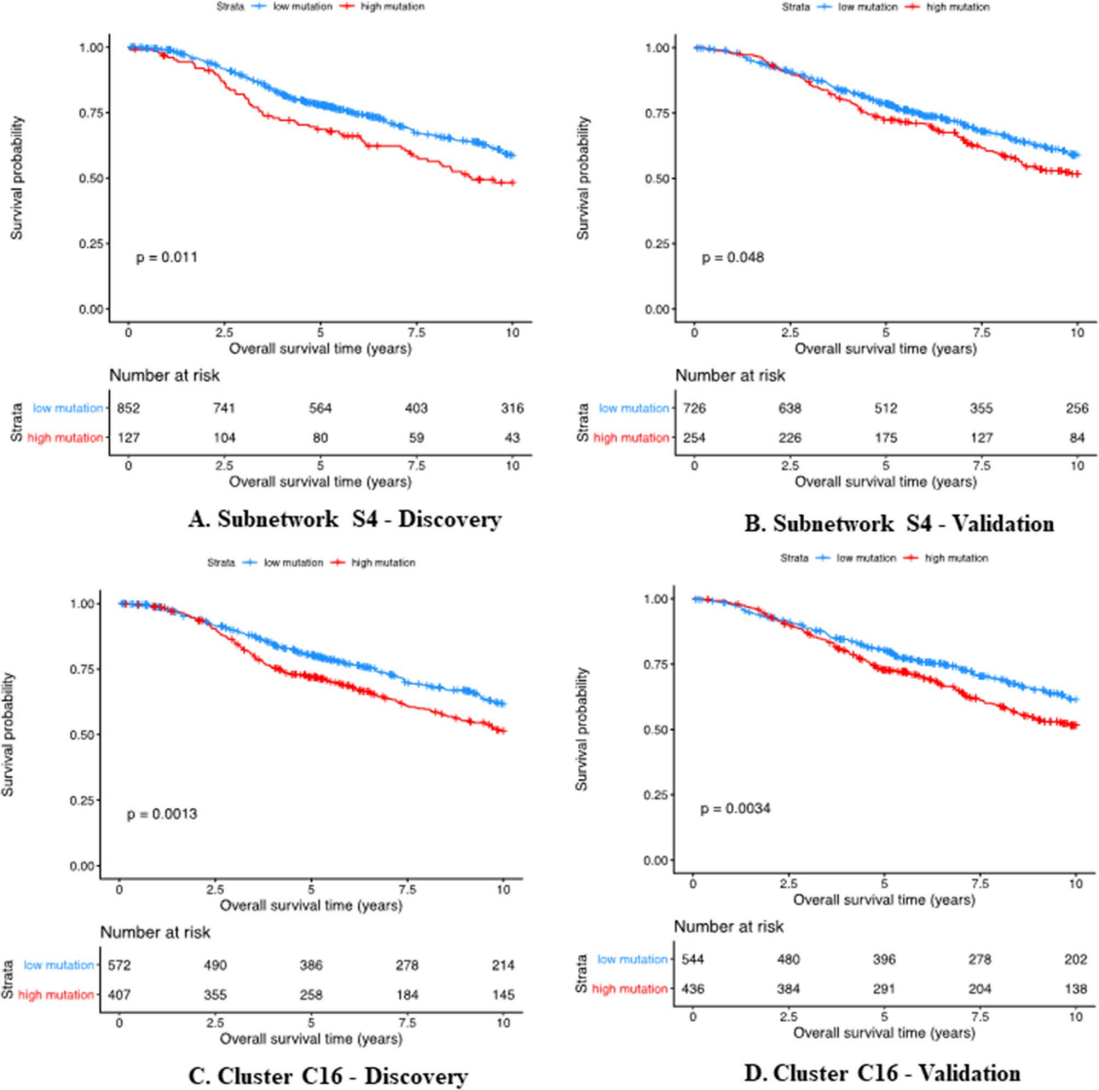
Kaplan–Meier survival analysis for the significantly mutated subnetwork S4 and cluster C16 based on real data. Survival plots based on the sample-specific mutation scores from the results of HotNet2 and ClusterONE. Patients were stratified into those with high mutation scores and those with low mutation scores based on the genes in the subnetwork S4 of HotNet2 in both the Discovery (**A**) and Validation (**B**) sets and gene cluster C16 of ClusterONE in both the Discovery (**C**) and Validation (**D**) sets. The blue curve shows the survival probability for the patients with low mutation scores and the red curve shows the survival probability for the patients with high mutation scores. The corresponding p-value is also shown in each plot.

Kaplan–Meier analysis using the sample-specific mutation score showed that the mutation patterns of the significantly mutated subnetwork S4 identified by HotNet2 is significantly associated with breast cancer survival in the Discovery set (Fig. 5A) and the Validation set (Fig. 5B). The same trend is also observed in the gene cluster C16 identified by ClusterONE in both the Discovery set (Fig. 5C) and the Validation set (Fig. 5D). The survival permutation *p* value for the significantly mutated subnetwork S4 and cluster C16 in the Discovery set and the Validation set are 0.002, 0.003, 0.005 and 0.006, respectively.

Generally speaking, the patients with high mutation scores are associated with worse outcomes. In other words, they have significantly shorter survival time than those with low mutation scores.

### Enrichment analysis

**Figure 6:**
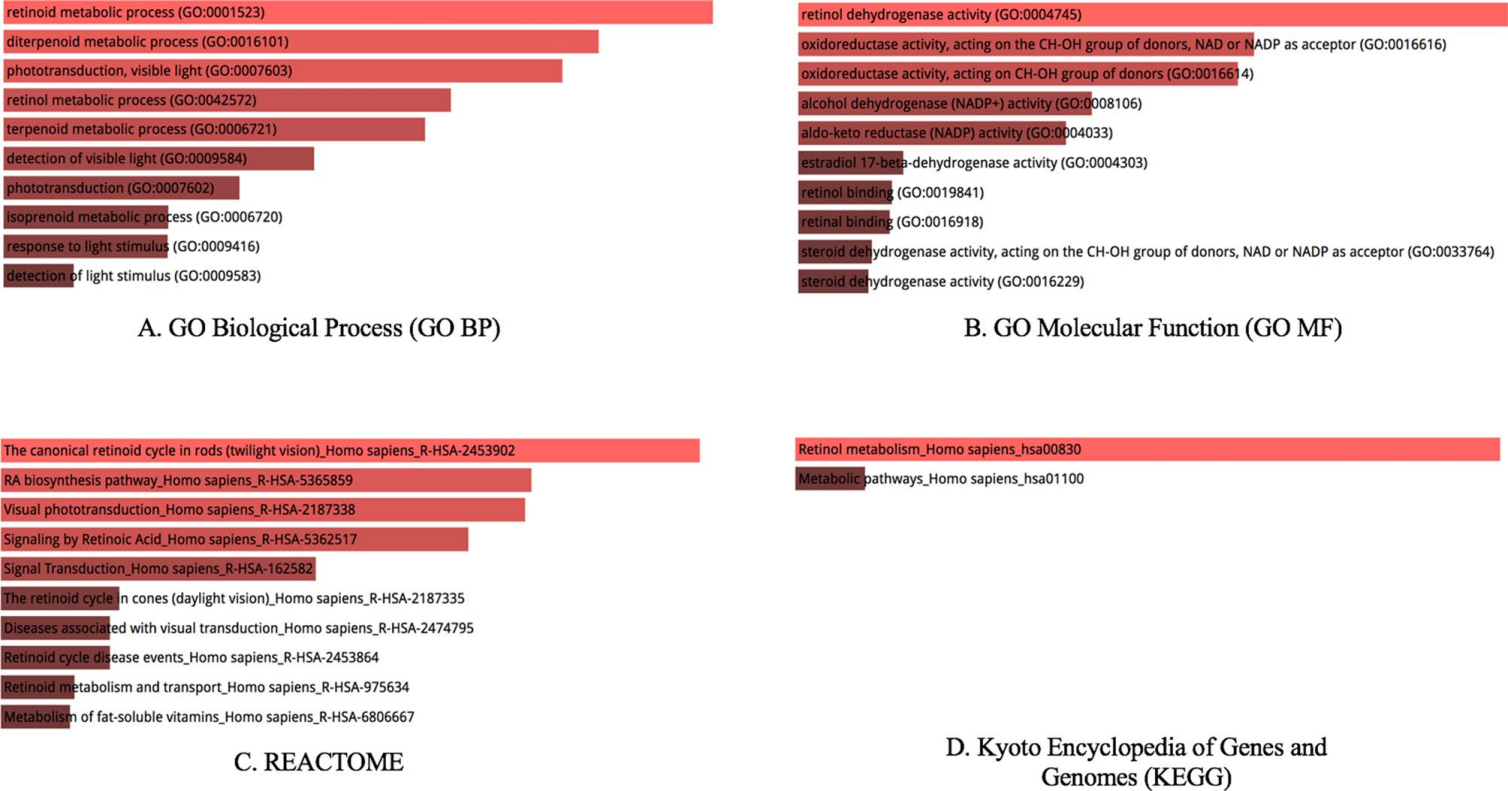
Enrichment analysis of the genes in significantly mutated subnetwork S4. Graph bars are sorted by *p* value ranking. The length of the bar represents the significance of that specific gene set. Light red colored bars have a *p* value < 1 × 10^–6^.

We further performed enrichment analysis of the seven genes *RDH5, RDH8, RDH10, RDH11, RDH12, RDH13, RDH14* via the Enrichr software. Our analysis revealed that the genes are significantly over-represented in retinoid metabolic biological process, retinol dehydrogenase activity molecular function and retinol metabolism (Fig. 6).

### Expression analysis of the identified genes

**Figure 7:**
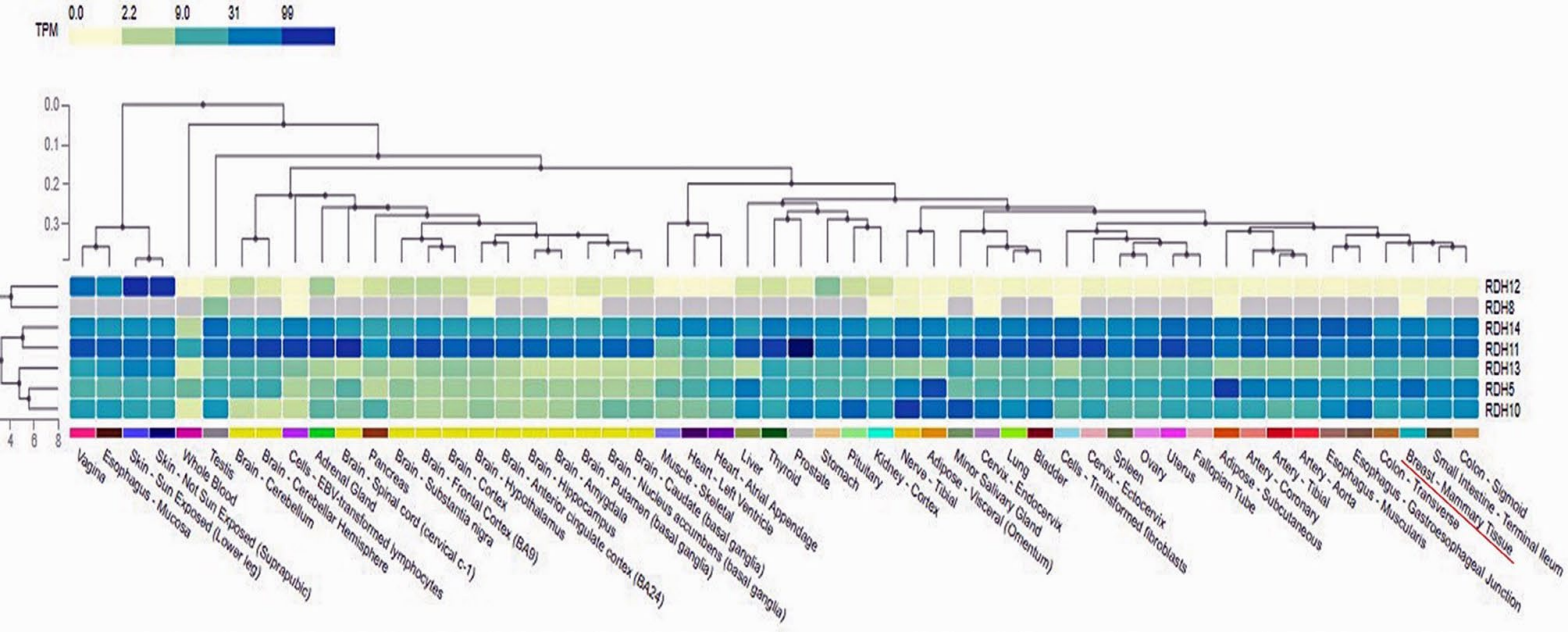
Expression patterns of the identify genes in normal human tissues from genotype-tissue expression (GTEx) portal. The Y-axis is the identified genes and X-axis is the human tissues in GETx portal.

To explore the expression patterns of the seven genes *RDH5, RDH8, RDH10, RDH11, RDH12, RDH13, RDH14* in normal tissues as well as breast cancer tissue, we first drew a heat map of the expression levels of the genes across normal human tissues from GTEx portal. As shown in Fig. 7, RDH5, RDH10, RDH11, RDH13 and RDH14 have a higher expression rate in breast tissue (underlined in red) while the expression rate of RDH8 and RDH12 appears to be rather lower in general. Furthermore, we also analyzed the expression levels of the genes at 50% and 90% quantiles in breast cancer samples from METABRIC and TCGA, respectively. Interestingly, as shown in the Table 4, the expression levels of the five highlighted genes (RDH5, RDH10, RDH11, RDH13 and RDH14) seems to be also higher compared to the other genes in breast cancer patients of the METABRIC data and the TCGA data.

**Table 4:** Expression of the identified genes in breast cancer samples form METABRIC and TCGA.

Gene	METABRIC (combined discovery and validation set)		TCGA	
	50% quantile	90% quantile	50% quantile	90% quantile
RDH5	6.332	7.225	61.210	315.634
RDH8	5.461	5.672	0	2
RDH10	7.076	8.840	1131.0	6645.4
RDH11	8.726	9.289	4528.0	7967.4
RDH12	5.394	5.562	5	16
RDH13	7.732	8.483	649.0	1218.8
RDH14	8.649	9.107	1240.0	1976.2

**Figure 8:**
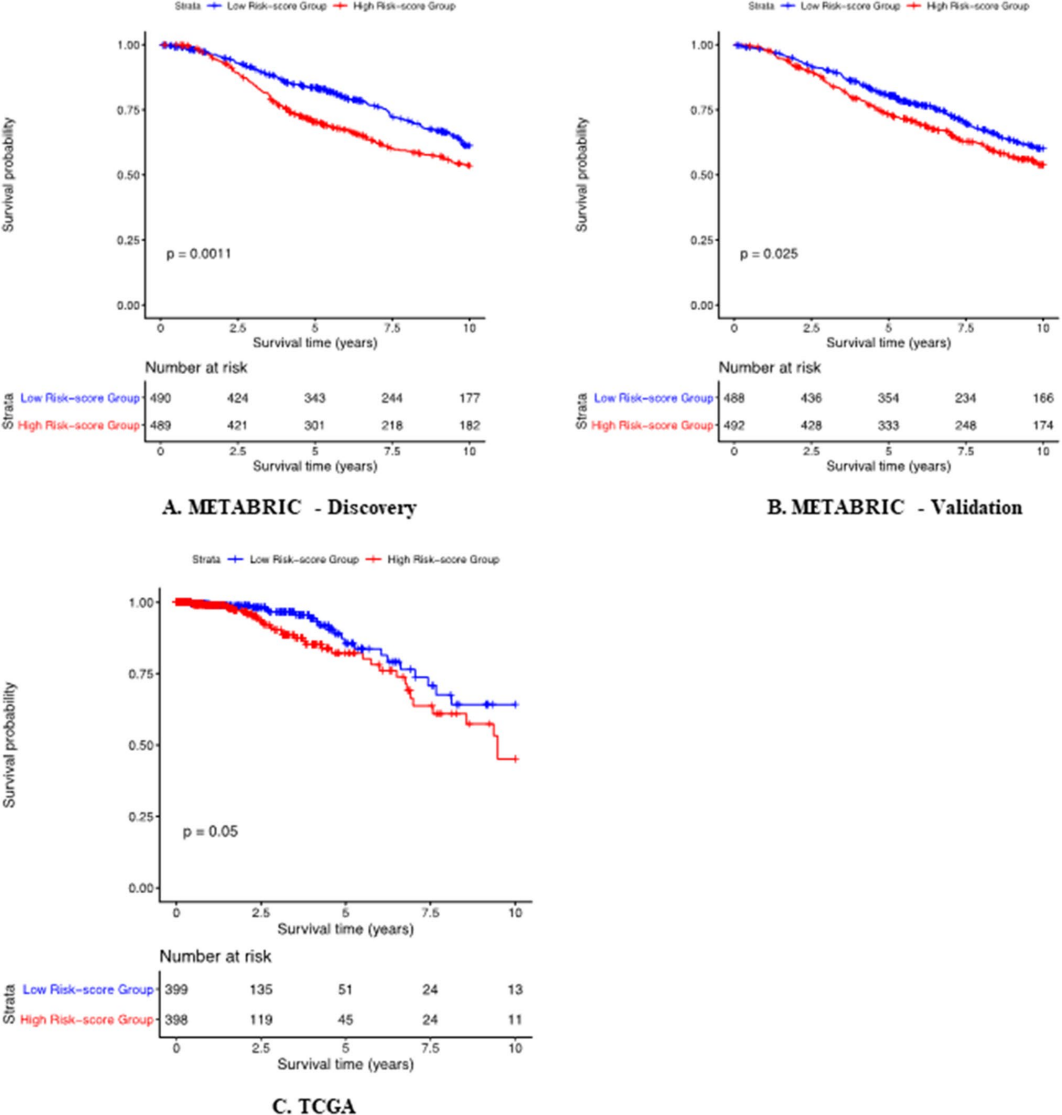
Kaplan–Meier survival analysis of the identified gene expression scores in METABRIC and TCGA data. Survival plots based on the sample-specific expression risk scores from METABRIC and TCGA data. Patients were stratified into those with high expression risk scores and those with low expression risk scores based on the identified genes in Discovery (**A**) and Validation (**B**) sets of METABRIC data and TCGA data (**C**).

Furthermore, we generated an expression risk score for each of the breast cancer patients in the METABRIC Discovery, Validation and TCGA sets using the seven genes *RDH5, RDH8, RDH10, RDH11, RDH12, RDH13, RDH14*. The KM survival showed the breast cancer patients with higher expression risk scores are significantly associated with worse outcomes while the breast cancer patients with lower expression risk scores are associated with good outcomes for METABRIC Discovery (Fig. 8A), Validation (Fig. 8B) and TCGA (Fig. 8C) sets, respectively, suggesting the robust efficacy of the identified potential survival biomarkers in breast cancer.

## Discussion

Many studies have found that retinoid receptors modulate various effects of retinoids, including estrogen metabolism in human breast carcinomas^29,30^. Interestingly, retinoids (such as vitamin A and its natural and synthetic analogs) have been used as potential chemotherapeutic or chemopreventive agents because of their differentiative, anti-proliferative, pro-apoptotic properties. Our analysis highlighted a number of retinol dehydrogenase (RDH) genes, belonging to the short-chain dehydrogenase/reductase (SDR) superfamily, to be significantly associated with breast cancer survival. To date, 47 SDR families, corresponding to at least 82 RDH genes, have been identified in human genome^31^. SDR superfamily constitute one of the largest enzyme superfamilies possessing more than 46,000 highly diverse members^32^ with only 15–30% sequence similarity^33^. The members of this enzyme family have been identified in all domains of life from bacteria to eukaryotes^32^. Based on structural differences in chain length, glycine binding motifs, and coenzyme binding motifs SDR families are grouped as classical SDRs and extended SDRs^33^. They play critical functional roles such as function in steroid hormone, prostaglandin and retinoid metabolism, signaling, and metabolism of xenobiotics such as drugs and carcinogens^31^. The functional role of SDR genes is highly related to the pathways contributed in breast cancer occurrence. Proliferation of hormone-dependent breast cancer is led by local production of estrogens^34^. Blockade of prostaglandin biosynthesis is considered as a prevention strategy against breast cancer^35^. Additionally, recent studies have delineated an association between retinol and breast cancer risk of ER-tumors^36^.

The members of SDR family are located in different part of the genomes on different chromosomes. RDH5, RDH8, RDH10, RDH11, RDH12, RDH13, RDH14 and SDR16C5, which we introduced as breast cancer survival biomarkers, are located in 12q13.2, 19p13.2, 8q21.11, 14q24.1, 14q24.1, 19q13.42, 2p24.2 and 8q12.1 genomic regions, respectively. They mostly participate in retinol dehydrogenation which is highly in consistence with He et.al findings which reported the dietary intake of β-carotene to be significantly associated with improved breast cancer overall survival^37^. It is noteworthy that dietary β-carotene is bio-converted to retinol (also known as vitamin A) which is essential for “the promotion of general growth, maintenance of visual function, regulation of differentiation of epithelial tissues, and embryonic development”^38^.

We acknowledge that there are many excellent algorithms for identifying mutated subnetwork. For example, Hofree et al. developed an elegant network-based stratification method to integrate gene network and somatic tumor variants for clustering patients into subtypes with different clinical outcomes^39^. The focus of this method is more on identification of tumor subtypes. Recently, Wiewie et al. applied different cluster methods, including the ClusterONE used in this study, to analyze network-based or tabular format data and found there was no universal best performer^40^. Furthermore, Batra et al. analyzed different pathway enrichment tools and they also showed that none of the methods consistently outperformed the others^41^. Hence, when we selected the algorithms for this study’s analysis, we have kept two issues in mind: (1) well-developed algorithms published on high-profile journals; (2) biologically-driven. The HotNet2 was specifically designed for identifying mutated subnetworks while the ClusterONE was specifically designed for identifying overlapping clusters. It is a common phenomenon that the same genes/proteins can be played roles in multiple pathways/function groups. One of key challenges in large-scale biomedical research is that there are many false positive discoveries. It is expected that the findings identified by multiple high-quality algorithms will be more robust than those identified in a single high-quality algorithm. Our findings showed that the group of genes we identified have potential prognosis for breast cancer based on their mutation burden.

Taken together, we believe that the role of our candidate genes in dehydrogenizing Vitamin A in addition to the mentioned roles of retinol can be evidence enough for the existence of a relationship between these genes and breast cancer survival.

## Conclusions

Since genes usually interact with other genes to execute their functions, gene networks can be modular and divided into subnetworks. It is reasonable to assume that clinically relevant mutations in breast cancer occur in closely interacting genes and breast cancer is an outcome of coordinated dysfunction of these closely connected subnetworks enriched with clinically informative cancer mutations.

We applied two well-established network analysis approaches to identify significantly mutated gene subnetworks using breast cancer copy number alterations, which included the approach to identify overlapping mutated subnetworks. This makes more biological sense because a gene can be assigned to multiple subnetworks and genes are usually involved in multiple pathways. Taken together, we found a significantly mutated yet clinically and functionally relevant subnetwork. The mutational pattern of the subnetwork is significantly associated with breast cancer survival. The genes in the subnetwork are significantly enriched in the retinol metabolism KEGG pathway.

## Supplementary Information

Supplementary Information.

## Data Availability

All the data used in the study are publicly available. Users can access the data based on our reference citations.
