# Supplementary material for: Identification of significantly mutated subnetworks in the breast cancer genome
Source: Sci Rep. 2021 Jan 12;11:642. doi: 10.1038/s41598-020-80204-5 (PMC7804148; doi:10.1038/s41598-020-80204-5)
Supplement: Supplementary file 1 — Supplementary Information. [file 41598_2020_80204_MOESM1_ESM.pdf]

Identification of significantly mutated subnetworks in the breast cancer genome

Rasif Ajwad, Michael Domaratzki, Qian Liu, Nikta Feizi, Pingzhao Hu

Additional File 1: Table S1. Parameters used to run Hotnet2

**Step 1: Influence Matrix Creation**			
**Dataset**	**Parameter Name**	**Value**	**Description**
Discovery	Edge swap constant, Q	115	The software performs $Q \times no\ of\ edges$ swaps
	Number of permutations	1000	Number of permuted networks to create
Validation	Edge swap constant, Q	115	The software performs $Q \times no\ of\ edges$ swaps
	Number of permutations	800	Number of permuted networks to create
**Step 2: Heat Score Generation**			
Discovery	Minimum Heat Score	1	The minimum score for the gene-specific mutation frequency
Validation	Minimum Heat Score	1	The minimum score for the gene-specific mutation frequency
**Step 3: Delta Selection**			
Discovery	Minimum Network Size	3	Minimum size of the connected components that should be returned
Validation	Minimum Network Size	3	Minimum size of the connected components that should be returned

Additional File 2: Table S2. Calculation of sample-specific mutation score

	KCNRG f = *0.3*	*TRIM13* f = *0.6*	*DGCR6L* f = *0.25*	*RIMBP3* f = *0.38*	*NOXA1* f = *0.2*	*RAC2* f = *0.4*	*ICMT* f = *0.5*	Mutation score
Sample 1	1	0	0	-1	0	0	1	1.18
Sample 2	0	1	-1	1	1	0	0	1.43
Sample 3	1	1	0	0	0	1	1	1.80
Sample 4	0	-1	1	0	-1	-1	-1	1.95
Sample 5	0	0	0	1	0	0	0	0.38
.	.	.	.	.	.	.	.	.
Sample n	-1	0	0	0	1	-1	0	0.9
